# Attenuated Stress Response to Acute Restraint and Forced Swimming Stress in Arginine Vasopressin 1b Receptor Subtype (Avpr1b) Receptor Knockout Mice and Wild-Type Mice Treated with a Novel Avpr1b Receptor Antagonist

**DOI:** 10.1111/j.1365-2826.2010.02070.x

**Published:** 2010-11

**Authors:** J A Roper, M Craighead, A-M O’Carroll, S J Lolait

**Affiliations:** * Henry Wellcome Laboratories for Integrative Neuroscience and Endocrinology, University of BristolBristol, UK; † Department of Molecular PharmacologyMSD, Newhouse, Motherwell, Scotland

**Keywords:** acute stress, vasopressin, Avpr1b receptor antagonist, hypothalamic-pituitary-adrenal axis

## Abstract

Arginine vasopressin (AVP) synthesised in the parvocellular region of the hypothalamic paraventricular nucleus and released into the pituitary portal vessels acts on the 1b receptor subtype (Avpr1b) present in anterior pituitary corticotrophs to modulate the release of adrenocorticotrophic hormone (ACTH). Corticotrophin-releasing hormone is considered the major drive behind ACTH release; however, its action is augmented synergistically by AVP. To determine the extent of vasopressinergic influence in the hypothalamic-pituitary-adrenal axis response to restraint and forced swimming stress, we compared the stress hormone levels [plasma ACTH in both stressors and corticosterone (CORT) in restraint stress only] following acute stress in mutant Avpr1b knockout (KO) mice compared to their wild-type controls following the administration of a novel Avpr1b antagonist. Restraint and forced swimming stress-induced increases in plasma ACTH were significantly diminished in mice lacking a functional Avpr1b and in wild-type mice that had been pre-treated with Avpr1b antagonist. A corresponding decrease in plasma CORT levels was also observed in acute restraint-stressed knockout male mice, and in Avpr1b-antagonist-treated male wild-type mice. By contrast, plasma CORT levels were not reduced in acutely restraint-stressed female knockout animals, or in female wild-type animals pre-treated with Avpr1b antagonist. These results demonstrate that pharmacological antagonism or inactivation of Avpr1b causes a reduction in the hypothalamic-pituitary-adrenal (HPA) axis response, particularly ACTH, to acute restraint and forced swimming stress, and show that Avpr1b knockout mice constitute a model by which to study the contribution of Avpr1b to the HPA axis response to acute stressors.

The cyclic nonapeptide arginine vasopressin (AVP) is synthesised centrally mainly in the paraventricular nucleus (PVN) and the supraoptic nucleus of the hypothalamus. Specific neuronal populations within the PVN can be subdivided into magnocellular and parvocellular neurones based on their organisation and function. Magnocellular neurones project to the posterior lobe of the pituitary gland where they release AVP and the structurally related peptide oxytocin into the bloodstream in response to physiological stimuli, including hypovolaemia or elevated blood sodium levels following haemorrhage or dehydration. Neuroendocrine parvocellular neurones project to the external zone of the median eminence where AVP enters the hypophysial portal circulation and is transported to the anterior lobe of the pituitary (1). Here, AVP together with corticotrophin-releasing hormone (CRH) modulates the secretion of adrenocorticotrophic hormone (ACTH) from pituitary corticotrophs into the peripheral blood supply as part of the response to stress by the hypothalamic-pituitary-adrenal (HPA) axis (1).

The actions of AVP are mediated through a family of closely related G protein-coupled receptors, which have been defined by their biological function, tissue distribution and pharmacological profiles: AVP receptor subtype 1a (Avpr1a), Avpr1b and Avpr2. Avpr1a is present in vascular smooth muscle and is responsible for the classical vasopressor action of AVP (2) and has many putative central roles (3–6); Avpr1b is primarily found in pituitary corticotrophs and modulates ACTH release, whereas Avpr2 controls water resorbtion in renal collecting ducts (7).

In response to a variety of homeostatic challenges, the HPA axis is activated as part of a complex endocrine and autonomic effort to control changes in the homeostatic environment. Various brain regions are activated as a result of such challenges, leading to a consolidated response from the PVN (8–10). Parvocellular ACTH secretagogues, AVP and CRH, act synergistically to promote ACTH release from the anterior pituitary gland. ACTH stimulates a rapid rise in blood glucocorticoid levels [cortisol in humans, corticosterone (CORT) in rodents]. There is evidence that magnocellular-derived AVP also participates in the control of ACTH release suggesting a direct influence of the hypothalamic neurohypophysial system on HPA axis function (1, 11). Glucocorticoids act on glucocorticoid receptors that are present in virtually all cells, to help control alterations in the homeostatic environment as a result of stress and also provide negative feedback at the pituitary, hypothalamus and higher brain centres. This ensures the stress response is transient by turning off activated systems once the stressor has ceased (12), preventing conditions associated with hypercortisolism. In unstressed rats, approximately half of CRH-expressing parvocellular neurones also express AVP, as measured by immunohistochemistry (13) and a large number of studies have shown that AVP plays a role, albeit perhaps minor compared to CRH, in the HPA axis response to the majority of acute stressors. With the discovery of ligands to pharmacologically manipulate Avpr1b (14–17), and the construction of Avpr1b knockout (KO) mice (18, 19), there is a growing impetus to investigate the contribution of Avpr1b to the HPA axis response to acute and chronic (or repeated) stressors. Our previous studies with Avpr1b KO mice revealed that Avpr1b is required for a normal HPA axis response to a variety of acute stressors [e.g. insulin-induced hypoglycaemia, lipopolysaccharide (LPS) and ethanol challenge (20, 21)]. In the present study we extend these observations to establish the role of Avpr1b in acute restraint and forced swimming stress using Avpr1b KO mice in conjunction with mice treated with a novel Avpr1b antagonist, Org (17).

## Materials and methods

### Animals

Adult (8–12 weeks old) mice (mix of C57BL/6J and 129 × 1/SvJ strains) (18) were group housed and maintained under a 12 : 12 h light/dark cycle (lights on 07.00 h) with controlled humidity (50%± 5%) at 21 ± 2 °C, with food and water available *ad lib*. Age-matched Avpr1b KO and wild-type male and female littermates resulting from heterozygous parental crosses (± for the Avpr1b mutation) were single housed 24 h before experimentation to facilitate injection and experimentation procedures. All female mice were used at random stages of the oestrous cycle. Animal experiments were performed between 08.00 and 14.00 h in accordance with Home Office regulations described by the UK Animals (Scientific Procedures) Act 1986, and were approved by the appropriate Bristol University Ethical Review Group.

### Acute restraint stress

Restrained mice were placed in a 50-ml plastic Falcon tube for 30 min before rapid decapitation. The tubes had up to ten small (approximately 5 mm) holes drilled around the sides of the tube and at its base to allow for sufficient ventilation. Control mice were briefly handled and returned to their home cage for 30 min before being killed. Restrained and handled mice were pre-treated with Avpr1b antagonist or sterile water as detailed below, with each experimental group containing five to seven mice. A previous study (22) has shown that a 30-min time point is suitable to assess both plasma ACTH and CORT hormone levels with this particular mild restraint stress.

### Acute forced swimming stress

Mice were placed in large glass beakers (1000/34D 5l, diameter 17 cm, height 27 cm; Bibby Sterilin, Stoke, Staffordshire, UK) filled to a depth of 15 cm with water (22 ± 2 °C) for 5 min then rapidly decapitated. Mice in control groups were handled briefly, returned to their home cage for 5 min, and then killed. As in the acute restraint experiment, mice were pre-treated with Avpr1b antagonist or vehicle, with each experimental group containing six to eight mice. Peak HPA axis activation after forced swimming stress, denoted by ACTH levels, was previously observed (22) immediately following stress, and hence was the time point chosen for this experiment. We have not reported CORT measurements in the present study because we do not know whether the single time point chosen would correspond to peak CORT levels and were unable to include additional time points as a result of constraints in animal numbers from our breeding colony. Tanoue *et al.* (19) working on another Avpr1b KO colony found that the ACTH response to forced swimming in Avpr1b KO and wild-type mice was not profoundly different between 2 and 10 min of continual stress, whereas CORT levels following 4 min of forced swimming were significantly lower than those obtained after 10 min of forced swimming in Avpr1b wild-type animals.

### Antagonist pre-treatment

Animals were pre-treated with either novel Avpr1b antagonist (Org) (17) or sterile nonpyrogenic water (vehicle). Doses of Org used were based on previous studies using the same compound (23, 24). Org solutions were freshly prepared on the day of injection in vehicle at doses of 10 and 30 mg/kg for restraint experiments; on the basis of the efficacy of these doses in this paradigm, we lowered the Org doses to 1, 3 and 10 mg/kg for forced swimming stress experiments. Animals were weighed the afternoon before experimentation and doses were calculated accordingly. Mice were injected with no more than 200 μl injection volume s.c. 2 h before commencing the stressor. Pre-treatment with Org or vehicle 2 h before stress is consistent with the time course of pre-treatment observed in a study by Craighead *et al.* (17); however, this differs from the time course given of 30 min in studies by Spiga *et al.* in rats (23, 24). We decided to pre-treat animals 2 h preceding the stressor, aiming to minimise the influence of injection stress in our animals, and to allow a more thorough absorption/uptake of antagonist into blood and tissues before excretion or blood clearance.

### Hormone analysis

Plasma obtained from the heparinised trunk blood of each animal was stored at −20 °C until analysis of ACTH and CORT concentrations by commercially available enzyme-linked immunosorbent assay and enzyme immunoassay kits (IDS, Tyne and Wear, UK), respectively. Each plasma sample was analysed in duplicate and all absorbance readings were performed at 450 nm with a Molecular Devices Versamax plate reader (Molecular Devices Corporation, Sunnyvale, CA, USA). All ACTH and CORT data are expressed as the mean ± SEM of each group and were analysed by three-way anova followed by a Tukey’s honestly significance difference post-hoc test using SPSS, version 18 (SPSS Inc., Chicago, IL, USA). P < 0.05 was considered statistically significant.

## Results

### The effect of Org on the HPA axis response to acute restraint stress

**Fig. 1:**
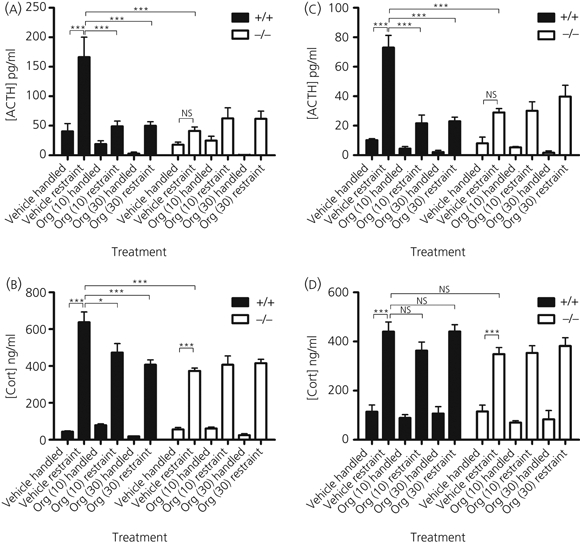
Plasma hormone responses to 30 min restraint stress after subcutaneous injection of 10 or 30 mg/kg Org or vehicle 2 h before the onset of stress in male (a, b) and female (c, d) wild-type (+/+) and arginine vasopressin receptor subtype 1a (Avpr1a) knockout (KO) (−/−) mice. All values are the mean ± SEM (n = 5–8 animals per group). Significance is denoted as: *P < 0.05–0.01, **P < 0.01–0.001, ***P < 0.001; NS, not significant. Wild-type plasma adrenocorticotrophic hormone (ACTH) levels (a, c) rose significantly in response to restraint stress versus handled controls in male and female mice. This rise in ACTH is reflected in the corticosterone (CORT) responses (b, d). Avpr1b KO plasma hormone levels also rose in response to restraint stress, with a marginally significant difference in ACTH data and a highly significant difference in CORT data versus vehicle treated, handled Avpr1b KO controls. Male and female Avpr1b KO mice had a considerably reduced ACTH and CORT response to restraint stress compared to wild-type mice subjected to the same treatment. Org injection significantly attenuated the stress-induced rise in ACTH at both 10 and 30 mg/kg doses in male and female experiments.

In the first set of experiments, wild-type and KO mice received s.c. injections of 10 and 30 mg/kg Org Avpr1b antagonist or vehicle before 30 min of restraint stress. Restraint increased the HPA axis hormone response in male and female wild-type mice (Fig. 1). Male plasma ACTH levels rose four-fold in wild-type stressed animals compared to the wild-type handled group (Fig. 1a: n = 5–7, P < 0.0001) and plasma CORT levels increased > 14-fold following stress in wild-type animals compared to the corresponding handled controls (Fig. 1b: n = 5–7, P < 0.0001). Such increases in ACTH and CORT response to restraint were also observed in female mice (Fig. 1c,d). Handled wild-type and KO mice in vehicle-treated, control groups had similarly low plasma ACTH and CORT levels. Although there was no significant increase in the ACTH response in either male or female Avpr1b KO mice subjected to restraint, the CORT response was significantly elevated (approximately 600 and 300% increase for males and females, respectively) compared to KO handled controls in male and female experiments (Fig. 1c,d). The plasma ACTH and CORT elevation in response to restraint found in male KO mice was 75% and 41% lower, respectively, compared to that observed in wild-type mice (ACTH, n = 7, P < 0.0001; CORT, n = 7, P < 0.0001). In female mice, the ACTH, but not CORT, response to restraint was attenuated in KO animals (ACTH levels decreased by 60%).

Injection of Avpr1b antagonist 2 h before the onset of 30 min of restraint had a profound effect on the stress-induced increase in plasma ACTH levels in male and female mice. The ACTH response to stress was reduced by 70% in wild-type male mice after pre-treatment with both 10 and 30 mg/kg antagonist (Fig. 1a; n = 7, P < 0.0001 at both doses). This trend was also observed in female mice (Fig. 1c), with Org reducing the stress-induced ACTH increase by approximately 70% at the two doses used (n = 6–7, P < 0.0001 at 10 and 30 mg/kg). This was not strictly matched in the plasma CORT responses to restraint stress because only male stress-induced CORT responses after antagonist pre-treatment were significantly reduced after antagonism; a decrease of 25% and 36% was seen at 10 and 30 mg/kg compared to vehicle treated wild-types, respectively (n = 7, P < 0.05 at 10 mg/kg and P < 0.0001 at 30 mg/kg; Fig. 1b).

Interestingly, restrained wild-type males and females pre-treated with Org (at both doses) show a reduction in plasma ACTH to levels approaching those observed in handled control mice. Following restraint, male and female KO plasma ACTH levels remain unchanged after Org pre-treatment (both doses) compared to vehicle-injected, restrained KO mice. This suggests that Org treatment has no antagonist activity on KO animals. Plasma ACTH levels in Org pre-treated, restrained wild-type mice duplicated that of the KO counterparts, indicating that the effect of Org closely corresponds to that of Avpr1b inactivation. The reduction of stress-induced plasma ACTH secretion to basal levels after injection with Org seen in the male and female ACTH data was not paralleled in the male or female CORT results, with wild-type restraint groups all being significantly higher compared to handled controls.

### The effect of Org on the HPA axis response to acute forced swimming stress

**Fig. 2:**
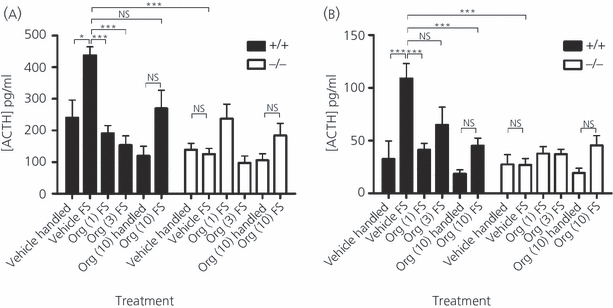
Plasma adrenocorticotrophic hormone (ACTH) responses to 5 min of forced swimming stress (FS) after subcutaneous injection of 1, 3 or 10 mg/kg Org or vehicle 2 h before the onset of stress in male (a) and female (b) wild-type (+/+) and arginine vasopressin receptor subtype 1a (AVpr1a) knockout (KO) (−/−) mice. All values are the mean ± SEM (n = 6–8 animals per group). Significance is denoted as: *P < 0.05–0.01, **P < 0.01–0.001 ***P < 0.001; NS, not significant. The forced swimming stress significantly elevated plasma ACTH levels in wild-type male and female mice with respect to handled control +/+ mice. Avpr1b mutant males and females displayed a profoundly reduced ACTH response to acute forced swimming stress compared to their wild-type counterparts in the same treatment group. Previous treatment of animals with 1 and 3 but not 10 mg/kg Org reduced male plasma ACTH levels to levels comparable with handled control mice. An equivalent reduction in plasma ACTH was seen in female wild-types with Org pretreatment. In each treatment group injected with Avpr1b antagonist, there was no significant difference between wild-type and Avpr1b KO mice ACTH levels in male and female cohorts.

The results obtained with respect to the peripheral injection of 1, 3 and 10 mg/kg Org, administered 2 h before 5 min of forced swimming in male and female Avpr1b wild-type and KO mice are shown in Fig. 2. Plasma from trunk blood collected immediately after swimming showed a rapid rise in wild-type mice plasma ACTH levels. Male vehicle-treated, wild-type control mice had plasma ACTH levels that increased 1.8-fold after swimming stress (Fig. 2a, n = 6, P < 0.05). Forced swimming also increased plasma ACTH levels in wild-type female mice compared to handled controls (Fig. 2b).

Avpr1b KO mice exhibit no increase in ACTH after forced swimming; the male and female KO ACTH response to stress were 71% and 75% lower than that of wild-type mice, respectively [males, Fig. 2a: Org injection at doses of 1 and 3 mg/kg attenuated swimming stress-induced ACTH release in male wild-type mice, reducing plasma ACTH by 56% (1 mg/kg, n = 6–8, P < 0.0001) and 65% (3 mg/kg, n = 6–8, P < 0.0001) with respect to vehicle-treated wild-type mice]. Interestingly, the reduction in plasma ACTH in wild-type, stressed mice seen after pre-treatment with 10 mg/kg failed to achieve significance, whereas the reduction following lower doses was significantly different. We observed a similar reduction of forced swimming-induced ACTH response in Org-injected female groups. Significant reductions in swimming stress-induced plasma ACTH levels were only noted after pre-treatment of female wild-type mice with 1 and 10 mg/kg.

As observed in the restraint stress data, the antagonist had no effect on KO plasma ACTH levels, which remained at the level of vehicle-treated swimming stressed KOs. On the other hand, wild-type mice pre-treated with Org had an attenuated ACTH response to forced swimming: ACTH plasma levels were decreased to a level similar to that for Org-treated stressed KO mice/vehicle-treated handled KO mice, as well as vehicle-treated handled wild-type mice.

The magnitude of ACTH response to forced swimming was smaller in stressed, wild-type female mice compared to the significantly larger response observed in similarly stressed male mice.

## Discussion

The present study employed both genetic and pharmacological manipulations to demonstrate that intact and functional Avpr1b are needed to mount a normal HPA axis response to both acute restraint and forced swimming stress. Previously, we have shown that mice lacking functional Avpr1b have a reduced HPA axis response to a number of ‘chemical’ or ‘physical-emotional’ stressors, including acute LPS challenge, ethanol injection (21), acute mild restraint, forced swimming stress, change of environment (22), insulin-induced hypoglycaemia (20) and acute antidepressant administration (25), compared to their wild-type counterparts. Avpr1b mutant mice generated by another group also show a reduced HPA axis response to acute forced swimming stress (19). There are a number of studies using the Brattleboro rat, a naturally occurring mutant strain lacking the ability to produce AVP, that emphasise the importance of AVP in driving the ACTH response to acute stress (26–30). The most recent of these studies (28) used Brattleboro rats in a variety of stress paradigms and concluded that the loss of AVP affects the HPA axis response to most (13 out of 18) of the stressors studied; there are specific stressors that exhibit a reduction in both ACTH and CORT (e.g. restraint), a reduction in ACTH only (e.g. forced swimming stress) and HPA stress responses that are not influenced by the loss of AVP (e.g. footshock). CRH levels remain unchanged in hypothalamic samples of unstressed Brattleboro rats (31), and the profound reduction in ACTH plasma levels following acute stress seen in this and our previous studies (20–22, 25) demonstrate that the loss of Avpr1b cannot be compensated for by CRH or other factors.

There is conflicting evidence with respect to the resting levels of ACTH and CORT in Battleboro rats and Avpr1b KO mice, with some studies reflecting reduced basal hormone levels [Brattleboro rats (32–34); Avpr1b KO mice (19)], whereas others report no difference [Brattleboro rats (26–30); Avpr1b KO mice (18) (CORT only)]. Our own previous observations suggest basal ACTH/CORT levels in Avpr1b KO mice are indistinguishable from wild-type levels, and we attribute this finding to resting levels of CRH or other ACTH secretagogues that are sufficient to maintain basal HPA axis activity. It is important to note that, in Brattleboro rats and Avpr1b KO mice, there may also be other ACTH secretagogues (e.g. oxytocin) released atypically, or that are present at altered levels, that may compensate for the loss of Avpr1b activity. Oxytocin which has a modest efficacy at Avpr1b is known to mediate ACTH release via Avpr1b (35, 36). This may feature as a compensatory mechanism in the Brattleboro rat, although it cannot compensate for the loss of Avpr1b in mouse mutants in this manner, as a result of the loss of Avpr1b itself rather than its cognate ligand. On the other hand, it has been reported that oxytocin at high levels can activate oxytocin receptors (Oxtr) present in pituitary corticotrophs to release ACTH (37) and that oxytocin could actively release ACTH in Avpr1b KO mice (37). Centrally administered oxytocin acting via the Oxtr reduces stress-induced CORT release in rats (38), and has been reported to exert anxiolytic effects in mice, possibly by modulating serotonergic neurone activity (39). Thus, oxytocin may have opposing actions on HPA axis activity depending on whether it acts on the central Oxtr or pituitary Avpr1b the activity of oxytocin at the central Avpr1b is not known. Our results obtained in wild-type animals pre-treated with an Avpr1b antagonist show a pronounced reduction in plasma ACTH after exposure to acute restraint and forced swimming stressors. The doses of Org used in the restraint (10 and 30 mg/kg) and forced swimming (1, 3 and 10 mg/kg) experiments generally show a similar reduction in the stress-induced ACTH release as a result of the effective blockade of Avpr1b. Exceptions to this trend appear in wild-type male and female mice exposed to swimming stress pre-treated with 10 and 3 mg/kg of Avpr1b antagonist, respectively. These groups just fail (P < 0.064) to achieve significantly lower plasma ACTH levels following forced swimming stress compared to vehicle-treated counterparts. The reason for these discrepancies are not clear; however, in both cases, there is a trend for the plasma ACTH levels to be lower than vehicle-treated swimming stressed animals and, indeed, these groups would have been statistically significant from their vehicle-injected counterparts had they each not included one ‘outlier’ (from a sample number of eight and seven, for the male and female groups, respectively), which had a value that was > 2 standard deviations away from the mean value. Antagonist administration in wild-type animals reduces the stress-induced ACTH release to levels akin to the stressed Avpr1b KO animals. As expected, Org injection in Avpr1b KO mice had no effect on stress-induced ACTH levels compared to stressed, vehicle-treated mutants. The reduction in ACTH levels after Org treatment in the present study is corroborated by findings observed with the antagonist in rats: ACTH levels are reduced after acute restraint stress and LPS immune challenge but not after noise stress (17, 23, 24). In keeping with the results using Org, the Avpr1b selective nonpeptide antagonist SSR149415 has also been shown to attenuate the ACTH response to restraint stress and ether exposure, yet the forced swimming, stress-induced ACTH response is unaltered in rats (14, 40). The specificity of SSR149415 has been questioned, with evidence of significant antagonism at the human Oxtr in addition to the human Avpr1b CHO cell lines expressing the recombinant receptors being noted (41). The high specificity of Org [human Avpr1b pKi 8.4 versus < 5 for all other human AVP receptors (17)] provides a precise tool, in addition to Avpr1b KO mice, by which to study Avpr1b. Org is also a highly effective Avpr1b antagonist in various rat models (17, 23, 24), while there are no profound differences in the AVP-binding profiles of rat and mouse Avpr1b (42), and the use of Org in mice has not been previously reported.

Our data on acute restraint highlight an apparent dissociation between plasma ACTH and CORT levels, when comparing wild-type versus Avpr1b KO mice or the vehicle treated wild-type versus Org treated mice. The removal of the Avpr1b contribution to the HPA axis response to acute stress by antagonist and genetic inactivation, markedly reduces plasma ACTH levels, although it has a less pronounced or marginal (if any, particularly in the case of our female CORT data) effect on CORT levels. It is possible that, because we only measured blood samples at a single time point, an incremental change in plasma CORT levels may have been missed. Dissociation between plasma pituitary ACTH and adrenal CORT secretions have been found in a number of acute and chronic stress studies in rodents (20, 22, 24, 26, 28, 43–45). Additionally, in analyses employing serial blood sampling techniques in rats, ACTH and CORT mismatches are often observed (24, 26, 28). Low levels of ACTH as seen in stressed Avpr1b KO animals may be sufficient to stimulate a full CORT response from the adrenal. In addition, a number of factors may influence adrenal glucocorticoid secretions (46) and it is clear that ACTH-dependent and independent, neural and immune variations influence adrenal sensitivity and glucocorticoid genesis or release (46, 47). Elevated plasma CORT levels with low ACTH levels as observed in the present study may also be a result of sympatho-adrenomedullary modulation of adrenal cortex sensitivity to ACTH; direct paracrine action or indirect refinement of adrenal cortical function by AVP on Avpr1a and Avpr1b (48) present in the adrenal medulla (49) suggests that adrenal cortex and medulla secretions may be influenced by the action of locally-produced AVP on the Avpr1a.

It is well established that the nature and severity of each stressor fundamentally alters how the HPA axis responds to each challenge, emphasising the complexity of how different stressors are interpreted, and the impact of numerous other influencing systems (e.g. immune activation) on HPA axis activation. Microarray data examining the transcriptional profiles in response to different acute stressors (LPS versus restraint stress in mice) (50) reveal that there is apparently little overlap in PVN gene expression, suggesting further scope for more detailed categorisation of stress effects by the genes activated. With respect to AVP, there is no obvious correlation between AVP influence (via Avpr1b activation) and specific categories of stressor (28). However, there are certain instances when the AVP system may drive the HPA axis; for example, Zelena *et al.* (28) note two stressors, immune challenge and insulin-induced hypoglycaemia, in which the contribution of the vasopressinergic system to HPA axis response in rats appears to be substantial. We have previously demonstrated the prominence of AVP/Avpr1b in the plasma ACTH and CORT responses to acute insulin-induced hypoglycaemia stress in mice (20). The restraint stress procedure used in the present study is milder than one we have previously employed (20) in which no difference was observed in the ACTH and CORT responses to acute exposure. A weaker HPA axis response to the less severe restraint stress permits the delineation of the vasopressinergic component driving the ACTH release, possibly because the salient ACTH secretagogue CRH, does not over-ride the subtle effects of AVP. Interestingly, restraint stress activates a number of immune-related molecules (e.g. interleukin-13) that are not activated by LPS challenge in mice (50). The activation of dissimilar immune pathways in response to different stressors may dynamically alter the HPA axis ACTH and CORT secretion profiles.

In the present study, we have noted some gender differences in the ACTH responses to acute restraint and forced swimming in wild-type mice (e.g. restraint-induced ACTH levels following Org pre-treatment are lower in males; the ACTH response to forced swimming is smaller in females). Because we did not examine ACTH or CORT levels at different stages of the oestrous cycle, we are unable to attribute such gender differences to changes in circulating sex steroids. However, gender differences in HPA axis regulation are not uncommon [in humans, females tend to display higher activity than males (51)], although this may be dependent on the nature of the stress (52) and, in rodents, such differences may be associated with the effects of sex steroids on AVP, oxytocin or CRH gene expression in the PVN (53, 54).

The rodent Avpr1b is found principally in anterior pituitary corticotrophs but is also detected in adrenal glands and the brain (55–58). The site at which Org impacts on ACTH secretion is likely to be the pituitary, although antagonism of central Avpr1b may also contribute to the overall effect of Org on the HPA axis response to acute stressors. Although Org has a relatively low brain penetration (17), we do not know the concentrations of antagonist that may be active at central sites *in vivo*. Avpr1b mRNA is present in brain regions that directly and indirectly innervate the PVN, such as the hippocampus and amygdala, and indeed the PVN itself (58). Although caution must be used when interpreting mRNA levels and extrapolating them to protein expression, the central Avpr1b gene expression leaves open the possibility that Avpr1b in higher brain centres that interpret specific pathways may modulate PVN activity. Intracerebroventricular injection of Org, or brain-region specific Avpr1b KOs/knockdowns, would likely demonstrate any potential effect of central Avpr1b.

We conclude that the elimination of vasopressinergic activity at Avpr1b either by genetic inactivation or pharmacological antagonism, results in an attenuated ACTH response to acute restraint and forced swimming stress. Comparable results produced by both Avpr1b KO and Org pre-treatment suggest that Org is a useful tool by which to further investigate the role of Avpr1b *in vivo*. Our results also highlight that Avpr1b KO mice used in our studies are unlikely to have developed substantial compensatory mechanisms to adjust for the loss of Avpr1b.
